# Human Brain Magnetic Resonance Imaging Studies for Psychiatric Disorders: The Current Progress and Future Directions

**DOI:** 10.31662/jmaj.2023-0167

**Published:** 2024-03-18

**Authors:** Jennifer Shi, Shinsuke Koike

**Affiliations:** 1 Department of Molecular Biology, Princeton University, Princeton, USA; 2 University of Tokyo Institute for Diversity and Adaptation of Human Mind, The University of Tokyo, Tokyo, Japan; 3 Center for Evolutionary Cognitive Sciences, Graduate School of Art and Sciences, The University of Tokyo, Tokyo, Japan; 4 The International Research Center for Neurointelligence, University of Tokyo Institutes for Advanced Study (UTIAS), Tokyo, Japan

**Keywords:** Magnetic resonance imaging (MRI), schizophrenia, psychiatric disorders, cross-disorder study, harmonization

## Abstract

With the prevalence of psychiatric disorders and the limitations of the diagnostic scheme and treatment options of these disorders, magnetic resonance imaging (MRI) studies play a significant role in uncovering the pathological basis of psychiatric disorders and potentially using biological markers in clinical settings. The use of MRI in clinical research has grown over the past three decades, and current MRI research continues to provide an avenue to guide the development of diagnostic approaches and therapeutic solutions. However, the current shortcomings of MRI studies derive not only from technical limitations (i.e., the range of contrasts that MRI probes or sensors can create) but also from confounding factors in the current methodological approaches of case-control studies for psychiatric disorders. Thus, by reviewing the recent literature on MRI research on psychiatric disorders, we explain the current progress and limitations of brain MRI methodologies used to study psychiatric disorders. We consider the growing use of cross-disorder methods to identify shared and disease-specific pathological features across psychiatric disorders. In addition, we need to outline healthy developmental and aging changes of the brain and investigate the disorder difference as a deviation of the trajectory. Although these methods have provided us with new insights, the demarcation between psychiatric disorders based on a definitive set of pathologies remains limited. This challenge of disease stratification is further complicated by the presence of multiple different sets of disorder pathologies *within* a single disorder and the different progressive timelines of different disorders. As such, we introduce the ongoing research projects in Japan, namely, the Brain Mapping by Integrated Neurotechnologies for Disease Studies (Brain/MINDS) and the Strategic International Brain Science Research Promotion Program (Brain/MINDS Beyond). These collaborative research initiatives across Japan use neuroimaging and travel-subject harmonization to conduct nationwide MRI studies capable of providing large-scale coherent results, which may address the current limitations of MRI psychiatric disorder research.

## 1. Introduction

**Figure 1.**
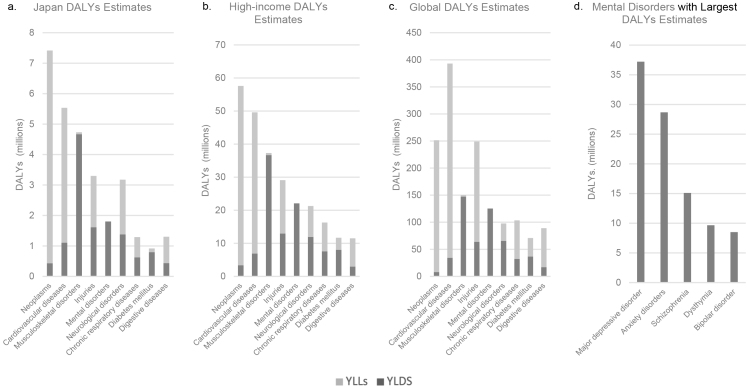
DALYs estimates from the Global Burden of Disease (GBD) 2019 study. DALYs estimates for nine significantly DALYs-affected diseases and events are shown for their global totals (a), high-income country totals (b), and Japanese totals (c). Total DALYs estimates split by years lived with disability (YLDs) and year of life lost (YLLs). (d) DALYs for mental disorders with the highest DALYs estimates.

Psychiatric disorders or mental disorders are a heterogeneous group of disorders that manifest through unusual psychological, cognitive, and behavioral patterns that cause distress or disability to an individual ^(1)^. Currently, approximately 9.7% ^(2)^ of the Japanese population and 12.6% ^(3)^ of the global population have psychiatric disorders. The global lifetime incidence rate is 4.7% ^(4)^. The Global Burden of Disease (GBD) 2019 study ^(5)^ provided estimates of the disability-adjusted life years (DALYs) for 369 diseases and injuries ^(6)^ as a part of its comprehensive review of the disease burden and scale of mortality worldwide ^(7)^. A DALYs estimate comprises two values, namely, the number of years lived with disability (YLDs) and years of life lost due to the disability (YLLs). Compared with the global 393 million DALYs estimate for cardiovascular diseases, which is the disease category with the greatest global DALYs estimate, the estimate for mental disorders amounted to 125 million DALYs (Figure 1) ^(8)^. In Japan alone, the DALYs estimate for mental disorders was 1.8 million DALYs ^(8)^. Within the category of mental disorders, the disorder with the greatest global DALYs estimate was major depressive disorder, followed by anxiety disorders, schizophrenia, dysthymia, and bipolar disorder ^(8)^.

In addition, it is imperative to recognize that approximately half of the first psychiatric disorder manifestations occur during the late teen years ^(9)^. Despite this early onset, individuals with these disorders are often less likely to seek medical help than those with nonpsychiatric diseases and afflictions ^(10)^. This reluctance to access medical or emotional support is largely due to the social stigma surrounding mental disorders, which has limited both the understanding and recognition needed for the proper treatment of mental disorders ^(11), (12)^. Thus, in addition to directly addressing the stigma surrounding these disorders on social grounds, the development of more precise risk assessment tools and treatment options when individuals seek help is needed. This comprehensive care is imperative for the psychological state, general physical health, and well-being of individuals with psychiatric disorders ^(13), (14), (15)^.

The diagnostic practice for psychiatric disorders primarily follows symptom-based clinical interviews by psychiatrists. Currently, the structural diagnosis scheme in psychiatry is guided by the Diagnostic and Statistical Manual of Mental Disorders (DSM-5) and the International Classification of Diseases (ICD-11), which both provide nosological approaches to psychiatric practice ^(16)^. However, various challenges still stand in the way of a psychiatrist’s ability to make an assured and confident diagnosis based on interviews. In addition to the complications of standardizing the psychiatric diagnosis procedure ^(17)^, the characteristics of psychiatric disorders are often difficult for patients to articulate and hard to be thoroughly understood by practitioners. Therefore, neurobiological markers with the ability to characterize mental disorders have an important potential to supplement symptom-based diagnosis.

Magnetic resonance imaging (MRI) has been used as a candidate research tool in elucidating the pathological characteristics of these disorders and applying the biological markers to clinical settings in the future ^(18)^. In this review, we intend to introduce a selection of recent and relevant MRI studies to highlight the current progress and limitations of MRI research in psychiatry. We illustrate how MRI methods can be used to inform the classification of psychiatric disorders, expand clinical treatment methods, and characterize disorder progression. Finally, we outline an ongoing neuroimaging project in Japan that seeks to overcome some of the limitations noted.

## 2. Current Progress in Human Brain MRI Studies for Psychiatric Disorders

### 2.1. Need for human brain MRI studies for psychiatric disorders

**Figure 2.**
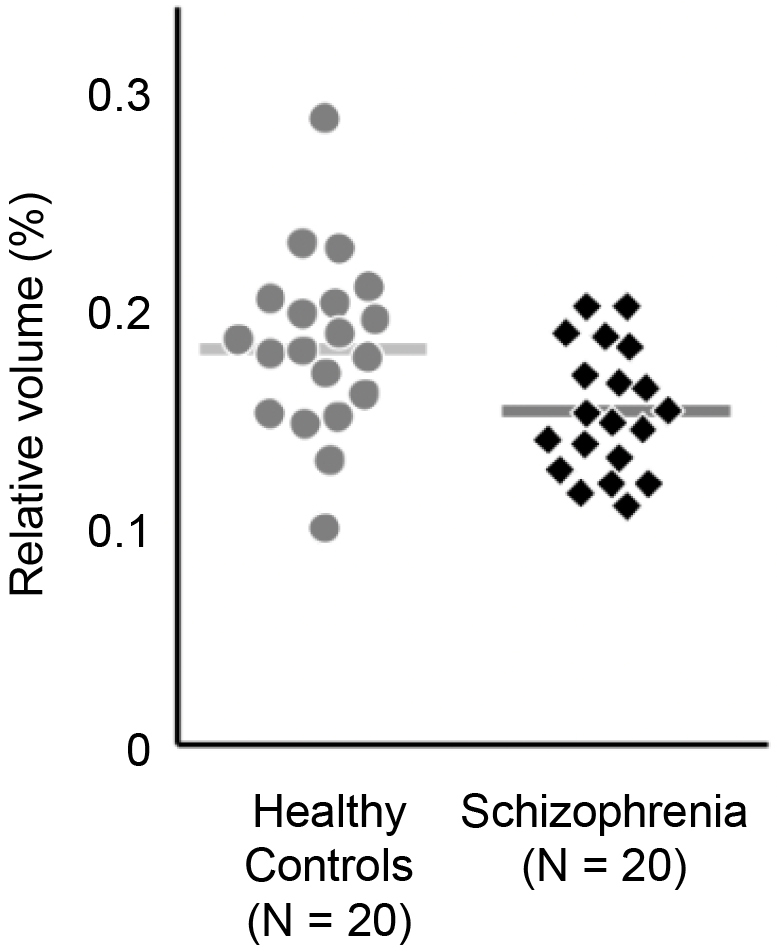
Finding from an example case-control MRI study for schizophrenia. This is an example finding from a case-control MRI study for psychiatric disorders that illustrates the group difference between individuals with schizophrenia and healthy controls for a brain feature, relative volumes in the right pars triangularis (PT) ^(20)^. Healthy controls exhibited an average higher relative volume of 0.183%. The schizophrenia group exhibited an average of 0.155%. Relative volume calculated by absolute volume/intracranial volume ×100.

Over 30 years have passed since MRI was first applied to clinical research of psychiatric disorders and brain pathophysiology began to be visualized ^(19)^. Figure 2 depicts the findings of a 2011 MRI study of schizophrenia conducted by Iwashiro et al., which compared the cortical volumes of the inferior frontal gyrus (IFG)―a region of the brain involved in language production and social skills―in 20 patients with schizophrenia and 20 healthy controls (age and gender matched) ^(20)^. Upon examining the relative volume of the pars triangularis of the IFG between the two groups, the schizophrenia group was observed to have a smaller IFG volume than that of the healthy control group ^(20)^.

An accumulation of such case-control studies has contributed to the elucidation of the pathological characteristics of various mental disorders. Advances in brain imaging research have contributed to the reduction of prejudice and discrimination against mental illnesses by highlighting the biological basis of these disorders. However, the diagnostic capabilities of MRI remain limited. For example, from the MRI study introduced above ^(20)^, although the aforementioned differences in cortical volume between patients with schizophrenia and healthy controls were found at the group level, the differences in volume were not discriminable between a healthy control and a participant with schizophrenia at the individual level. The subjects with the largest and smallest cortical IFG volumes were both parts of the control group. Therefore, IFG cortical volume alone would not be sufficient as a diagnostic criterion for schizophrenia.

### 2.2. Current progress and limitations of MRI methodologies used to elucidate brain pathology: cross-disorder psychiatric studies

A limitation of case-control studies is the isolation of the findings to the specific disorder of the study ^(21)^. Namely, case-control studies attribute their pathological findings to the disorder of focus, which potentially discounts the possibility that these pathologies are shared across various other psychiatric disorders or even other neurological disorders, and thus, it is not a viable marker for one disorder. For example, individuals with autism spectrum disorder have decreased IFG volumes compared with healthy individuals ^(22)^, which was also observed in the schizophrenia study above. Neuroimaging and genetic studies have also proposed a shared neural basis between autism and schizophrenia ^(23)^. Without proper synthesis of findings from more than one disorder, the results from case-control studies may lead to the mischaracterization of transdiagnostic pathology as disorder-specific pathology and inaccurate frame distinctions between different disorders.

The Enhancing NeuroImaging Genetics through Meta-Analysis (ENIGMA) consortium has compiled case-control studies from around the world into a 30,000+ person population sample with psychiatric and neurodevelopmental disorders of interest ^(24)^. Notably, an ENIGMA meta-analysis of the transdiagnostic covariance between six different psychiatric and neurodevelopmental disorder groups (ASD, ADHD, MDD, SCZ, BD, and OCD) yielded a set of pathologies and alterations not observed in healthy controls ^(24)^. In this analysis, shared thickness alterations were identified in the lateral and ventral temporal and frontal lobes of the brain, particularly in the regions of sensory and attention-level networks across the disorders. Therefore, the measured pathological differences *between* psychiatric disorders were often of very little significance ^(24)^. The heterogeneity in highly related diagnostic categories (e.g., schizophrenia and bipolar disorder) was not observed. These findings demonstrate that many of the morphological alterations observed in one psychiatric disorder in a case-control study are often not specific to this disorder but rather observe across multiple disorders.

As a result, a growing number of MRI studies of mental disorders have been developed to encompass cross-disorder studies. This study approach offers an examination of shared morphological abnormalities across psychiatric disorders. Hence, cross-disorder MRI studies require multiple participant populations of different disorders to elucidate the shared morphology, connectivity networks, or neurotransmitter circuits of these disorders ^(25), (26), (27)^. For example, using the longitudinal neuroimaging cohort from adolescence to young adulthood (IMAGEN) ^(26)^, researchers explored the presence of a neuropsychological factor (referred to as the NP factor) that is shared across multiple psychiatric disorders. This NP factor was determined to characterize a shared delay in prefrontal cortex development and hyperconnectivity with “prefrontal-related neural circuits” ^(26)^ in multiple psychiatric disorders. Another research identified other circuits (e.g., midbrain dopaminergic system-related circuits ^(28)^) that display connectivity changes observed similarly across multiple psychiatric disorders. As such, both cross-disorder structural MRIs and studies of connectivity have elucidated pathological similarities across disorders.

**Figure 3.**
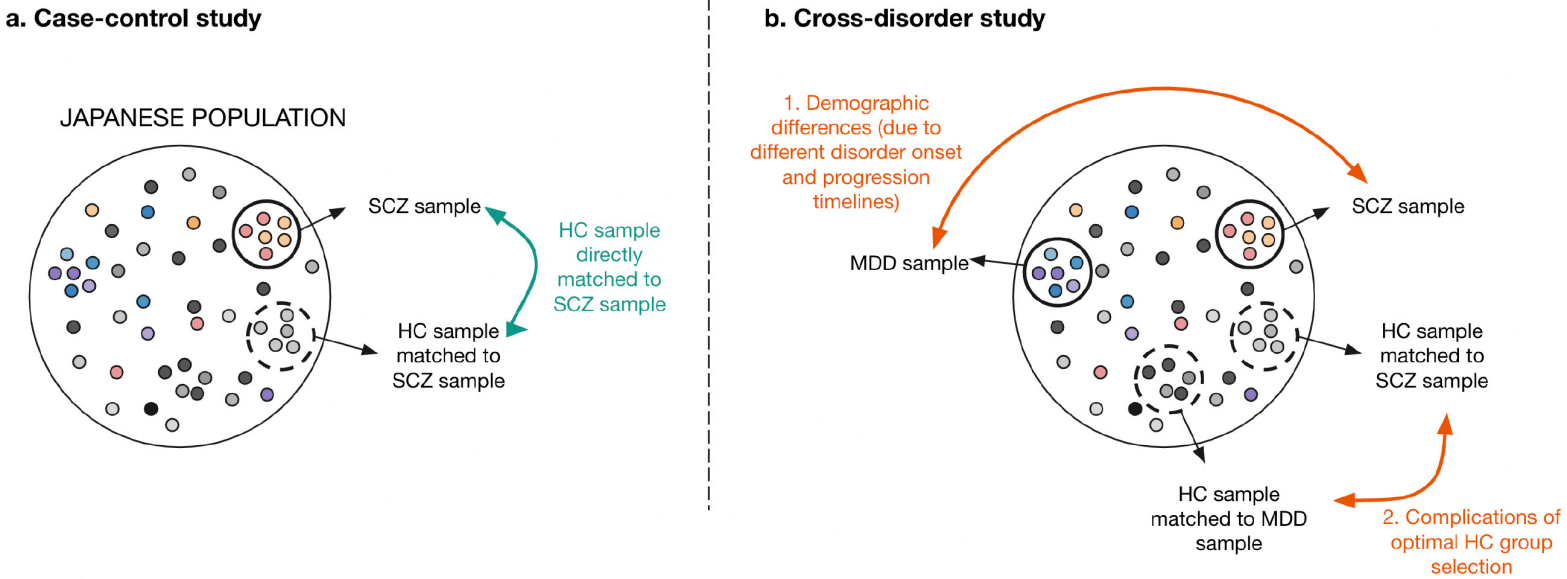
Sample population confound in a cross-disorder study. (a) In a case-control study, healthy control (HC) samples are generally matched for demographic characteristics (e.g., sex, age, and socioeconomic status) to the schizophrenia (SCZ) samples, which limits to control the potential effect of demographic-based confounders on the biological measurements (e.g., brain structural characteristics). (b) In a cross-disorder study (e.g., SCZ vs. major depressive disorder [MDD]), which is the first study conducted from a combination of datasets in case-control studies, the demographic characteristics of the two psychiatric disorder groups and the two matched control samples may have different demographic features because of different peak ages at onset and progression timelines between the disease groups.

However, cross-disorder studies are often not approached with sufficient attention to sample population selection. Because mental disorders have different onset ages and progressive tracks ^(21)^, finding comparable sample populations (i.e., groups with similar demographics or backgrounds) across psychiatric disorders is challenging. Using case-control studies as a counterpoint, the healthy control population of a study can be directly matched to the sample disorder of the focus population (Figure 3a). However, in cross-disorder studies, there is more likely to be incongruence between the sample groups of each disorder because the disorders develop and progress at varying timescales and show varying prevalence in different demographic populations. For example, the healthy controls studied for major depressive disorder are more likely to be older and female compared with those for schizophrenia because of discrepancies in disease population prevalence and progression ^(8)^. These discrepancies also complicate the selection of a healthy control group that is demographically comparable to more than one psychiatric disorder (Figure 3b). This limitation needs to be considered when demographic variables are known to affect biological markers, such as brain measurements. In this case, large samples with a wide age range (e.g., more than 1,000 HCs from those aged 15-60 years) are needed to determine the deviation from normative healthy development and aging ^(18), (29), (30), (31)^. These complications do not discourage the use of the cross-disorder study approach, but instead, they emphasize the need for better qualitative controls capable of considering both clinical disorder progression and additional confounding demographics.

Although these similarities in brain pathology across disorders are useful for transdiagnostic criteria, they also present a roadblock in using MRIs in the diagnostic setting for individual disorders. Cross-disorder studies emphasize the presence of several pathologies in one disorder while simultaneously revealing that the presence of a single neural basis can be related to several disorders. This pathology-disorder dilemma prevents the construction of precise diagnostic criteria for each disorder. Furthermore, the outward-presenting symptoms of a patient often do not fall cleanly into the criteria of one disorder, thus explaining the presence of differential diagnoses for psychiatric disorders within DSM-5. For example, schizophrenia has differential diagnoses, including major depressive disorder with psychotic features, delusional disorder, and obsessive-compulsive disorder ^(1)^. Therefore, it may not be surprising that neurological pathologies observed in MRI studies are also shared across disorders. Thus, it is necessary to consider how MRI methods can be used to elucidate potential heterogeneities more clearly between disorders and evaluate their dependability and consistency within case-control studies.

### 2.3. Current progress and limitations of MRI clinical applications: targeting the neurochemical basis of psychiatric disorders for treatment

The extensive heterogeneous group of neurochemical factors that contribute to psychiatric disorders complicates approaches used to determine the most effective clinical treatment. We use the dopamine hypothesis in schizophrenia as an example ^(32)^. The initial hypothesis of hyperdopaminergia in schizophrenia is based on the clinical findings that hallucinations and delusions are ameliorated by dopamine antagonists ^(32)^. These findings are supported by positron emission tomography (PET), which provides a mechanism for visualizing dopamine hyperactivity in the striatum and for examining the effects of therapeutic agents on activity. However, the dopamine hypothesis alone is not sufficient to explain the symptoms of schizophrenia. For example, one-third of individuals with schizophrenia fulfill the treatment resistant criteria ^(33)^ and show little to no improvement in symptoms after being prescribed dopamine-antagonist medication. Thus, further research on how dopamine is dysregulated across different regions and networks of the brain and how other neurochemicals (i.e., glutamatergic and GABAergic systems) contribute to the symptoms is needed to supplement the explanation for the neurochemical basis of schizophrenia ^(33)^.

The technical challenges of obtaining in vivo neurochemical data of the human brain limit the ability to understand the neurochemical mechanisms of psychiatric disorders. PET is one of the main tools used for studying neurotransmitter behavior, pathways, and imbalances in vivo ^(34)^. MRI has served to supplement the findings from PET scans and can come in the form of pharmacological MRI (ph-MRI), which examines the signal changes in the brain before and after the administration of a psychiatric drug. ph-MRI is often used to examine drugs targeting the dopamine and glutamate pathways, and thus, it provides a method to identify the region of drug action fingerprinting ^(35)^, informing us of drug development and efficacy. PET scans must confirm that the signal changes observed in ph-MRI are related to the neurotransmitter systems and pathways of interest.

In addition to these methods, standalone MRI methods capable of studying neurochemistry are in development. For example, researchers have evaluated the potential of MRI measurements of neuromelanin (NM-MRI) as a “proxy measure of brain dopamine in psychiatric disorders” ^(36)^. An experiment comparing unmedicated patients with schizophrenia and healthy controls revealed higher levels of neuromelanin accumulation on MRI in the former group. This was found to be correlated with both higher dopamine release in nigrostriatal neurons and the severity of schizophrenia. Furthermore, earlier NM-MRI studies have already revealed the potential of using the signal alterations of neuromelanin in the substantia nigra and locus coeruleus to distinguish patients with psychosis ^(36)^ from patients with depression and healthy controls ^(37)^.

### 2.4. Current progress and limitations in characterizing disorder progression and developmental trajectory using MRI

For every psychiatric disorder experiment, an understanding of how the disorder progresses, such as when pathological changes occur and how symptoms reflect these changes, is invaluable. Without consideration of disorder progression, MRI analysis of a subject group lacks the context needed to make reliable pathological conclusions as individuals may be at drastically different stages of disorder progression. However, the characterization of progression presents with various challenges. Notably, one challenge is rooted in the prevalence of psychiatric disorder onset in adolescence and young adulthood. As the adolescent brain is simultaneously changing through the course of normal development, characterizing disorder progression requires the additional task of distinguishing between normal developmental changes and disorder-related changes ^(38)^.

Longitudinal cohort studies play a crucial role in examining psychiatric disorder progression. The hippocampus has been an area of interest for these studies due to findings of smaller hippocampal volumes across multiple disorders ^(39)^. We examined two longitudinal MRI studies that both revealed reductions in hippocampal volumes over time (although in different subregions), namely, one in individuals with early psychosis (onset mean age: 21 years) ^(40)^ and the other in psychotic experience-presenting adolescents (onset mean age: 12 years) ^(41)^. The study on patients with early psychosis used a two-year timeline. At the onset of the experiment, the researchers identified a deficit magnitude in the anterior hippocampus size in the early psychosis group compared with healthy controls. At the two-year follow-up, this deficit persisted with the same degree of difference, although the average hippocampal size increased equally between both groups ^(40)^. The study examining psychotic experience-presenting adolescents followed a six-year time course and found a deficit in the right hippocampus volume after six years. However, contrary to the first study, the initial volume between the psychotic-presenting and control groups was approximately the same, so the observed growing deficit was due to psychotic experience-presenting adolescents exhibiting no increase in hippocampal volume. Only the volume of the healthy control group was observed to increase after experiment onset ^(41)^. These variations in findings from each respective time course highlight the variability of disorder progression or the effect of the differences in imaging age on the findings of morphological change.

Furthermore, meta-analysis findings from an examination of multiple longitudinal MRI studies of one psychiatric disorder often reveal varying levels of consensus for different brain regions. For example, a meta-analysis of 27 longitudinal MRI schizophrenia papers revealed both consensus in gray matter reduction in multiple regions of the brain and minimal changes in the whole brain volume over time. However, findings on longitudinal alterations in lateral ventricular size, for example, have yielded mixed results across studies ^(42)^. Discrepancies between studies were most pronounced between the respective hypotheses presented by researchers on the correlation between a particular brain pathology and measures of symptom severity, positive or negative symptom scores, or specific cognitive deficits ^(42)^. Thus, it is necessary to clearly identify the specific changes that are relevant and important to characterizing the prognosis, development, and specific traits of a disorder.

## 3. Ongoing Project in Human Brain MRI Studies for Psychiatric Disorders

To overcome the limitations above, the Japan Agency for Medical Research and Development (AMED) has funded neuroimaging studies for neuropsychiatric disorders. Brain Mapping by Integrated Neurotechnologies for Disease Studies (Brain/MINDS) ^(43)^ and the Strategic International Brain Science Research Promotion Program (Brain/MINDS Beyond) ^(44)^ have promoted a domestic collaborative research system in Japan. Participating institutions acquire neuroimaging data using a standardized protocol for high-resolution MRI scanners and harmonize the data using traveling subject (TS) measurements, in which the same subject undergoes MRI scans at different institutions to diminish machine- and protocol-derived differences. The TS harmonization method is relatively effortful for participants and researchers compared with other harmonization methods such as mathematical and statistical models, but the difference between participating sites diminishes up to a test-retest scan level ^(45)^. Brain/MINDS Beyond has already obtained more than 700 TS scans from 80 participants, which are shared and used for database construction and development of data preprocessing. Data, including disease groups and developed technologies, are shared within the participating institutions and are made publicly available without restriction after the completion of the project (FY2024~). The projects also provide technical information and education courses for young researchers and students. Tutorials related to the MRI projects are held every year, and many of them are available in the form of YouTube videos (https://hbm.brainminds-beyond.jp/ja/).

## 4. Conclusion

MRI research has been proven to be a valuable tool for understanding the morphological, neurochemical, and developmental features of various psychiatric disorders. Cross-disorder studies, particularly when studied within the context of meta-analyses, have been crucial in identifying similarities in cortical volume and thickness, neurocircuit networks, and biochemical markers present across psychiatric disorders. However, the applicability of these findings to diagnostic and clinical settings remains limited. MRI research continues to grapple with the challenges of identifying and characterizing a set of pathologies that are capable of comprehensively defining a psychiatric disorder and thus can be used in the clinical setting. In addition, subdisorder stratifications make optimal MRI-based diagnosis and targeted therapeutic treatment more elusive. However, multimethod diagnosis using traditional DSM-5 interview-based criteria and MRI in tandem could be a point of entry into using imaging for diagnostic procedures.

Longitudinal MRI studies have contributed to elucidating the timeline of disorder progression, demonstrating variations in onset age, and examining the rate and nature of alterations across psychiatric disorders. These findings highlight the potential for selection bias when selecting sample populations for cross-disorder studies. The level of confidence in the comparability of two different psychiatric disorder populations is highly reliant on the demographic differences inherent to the respective disorders, and this makes selection of a healthy control group to match multiple diseases challenging. Thus, it is imperative to pay closer attention to population demographics when comparing across disorders to achieve more cohesive and reliable results. Moving forward, deep learning algorithms trained on MRI scans have been used as a potential mechanism for pathology-based diagnoses ^(46), (47)^. Although findings reveal ranging success in MRI-based diagnoses among psychiatric disorder populations, most findings are in the preliminary stages. Notably, diagnostic accuracy greatly drops for early-stage disorder populations ^(47)^. However, these emerging methods open a promising new avenue for MRI research.

Finally, the growth of meta-analysis methodology and large-scale collaborative MRI studies, such as the Brain/MINDS project, will be another avenue for expanding coherent and cross-validated pathological findings across research institutions. TS harmonization will be valuable in confirming multiinstitution findings, and large-scale database results can provide a platform for further meta-analysis.

## Article Information

This article is based on the study, which received the Medical Research Encouragement Prize of The Japan Medical Association in 2022.

### Conflicts of Interest

None

### Acknowledgement

This review was supported by the Japan Society for the Promotion of Science (JSPS)/MEXT KAKENHI (JP23H03877 and JP22H05212), Japan Agency for Medical Research and Development (AMED; JP18dm0307004 and JP19dm0207069), and Japan Science and Technology Agency Moonshot R&D Grant Number JPMJMS2021. This study was also supported by the World Premier International-International Research Center for Neurointelligence (WPI-IRCN).

## References

[1] American Psychiatric Association. Diagnostic and Statistical Manual of Mental Disorders. 5th ed. Washington DC: American Psychiatric Association; 2013.

[2] Dattani S, Rodés-Guirao L, Ritchie H, et al. Mental health. Our world data [Internet]. 2023 Jun 20 [cited 2023 Jun 26]. Available from: https://ourworldindata.org/mental-health

[3] GBD 2019 Mental Disorders Collaborators. Global, regional, and national burden of 12 mental disorders in 204 countries and territories, 1990-2019: a systematic analysis for the Global Burden of Disease Study 2019. Lancet Psychiatry. 2022;9(2):137-50. 35026139 10.1016/S2215-0366(21)00395-3 PMC8776563

[4] The Lancet. Global burden of disease: GBD cause and risk summaries: mental disorders-level 2 cause [Internet]. 2019 [cited 2023 Jun 26]. Available from: https://www.thelancet.com/gbd/summaries

[5] World Health Organization. Disability-adjusted life years (DALYs) [Internet]. [cited 2023 Jun 27]. Available from: https://www.who.int/data/gho/indicator-metadata-registry/imr-details/158

[6] Vos T, Lim SS, Abbafati C. Global burden of 369 diseases and injuries in 204 countries and territories, 1990-2019: a systematic analysis for the Global Burden of Disease Study 2019. Lancet. 2020;396(10258):1204-22. 33069326 10.1016/S0140-6736(20)30925-9 PMC7567026

[7] Global Burden of Disease (GBD). Institute for health metrics and evaluation [Internet]. 2014 Mar 29 [cited 2023 Jun 27]. Available from: https://www.healthdata.org/gbd

[8] Global Burden of Disease Collaborative. Network. Global burden of disease Study 2019 (GBD 2019) results. Institute for Health Metrics and Evaluation [Internet]. 2019 [cited 2023 Jul 7]. Available from: https://vizhub.healthdata.org/gbd-results

[9] Kessler RC, Amminger GP, Aguilar‐Gaxiola S, et al. Age of onset of mental disorders: a review of recent literature. Curr Opin Psychiatry. 2007;20(4):359-64. 17551351 10.1097/YCO.0b013e32816ebc8c PMC1925038

[10] Radez J, Reardon T, Creswell C, et al. Adolescents’ perceived barriers and facilitators to seeking and accessing professional help for anxiety and depressive disorders: a qualitative interview study. Eur Child Adolesc Psychiatry. 2022;31(6):891-907. 33502596 10.1007/s00787-020-01707-0 PMC9209355

[11] Thornicroft G, Brohan E, Rose D, et al. Global pattern of experienced and anticipated discrimination against people with schizophrenia: A cross-sectional survey. Lancet. 2009;373(9661):408-15. 19162314 10.1016/S0140-6736(08)61817-6

[12] Ando S, Nishida A, Usami S, et al. Help-seeking intention for depression in early adolescents: associated factors and sex differences. J Affect Disord. 2018;238:359-65. 29908475 10.1016/j.jad.2018.05.077

[13] American Psychiatric Association. Stigma, prejudice and discrimination against people with mental illness [Internet]. [cited 2023 Jun 26]. Available from: https://www.psychiatry.org:443/patients-families/stigma-and-discrimination

[14] Thornicroft G, Mehta N, Clement S, et al. Evidence for effective interventions to reduce mental-health-related stigma and discrimination. Lancet. 2016;387(10023):1123-32. 26410341 10.1016/S0140-6736(15)00298-6

[15] Koike S, Yamaguchi S, Ojio Y, et al. A randomised controlled trial of repeated filmed social contact on reducing mental illness-related stigma in young adults. Epidemiol Psychiatr Sci. 2018;27(2):199-208. 27989255 10.1017/S2045796016001050 PMC7032789

[16] First MB, Gaebel W, Maj M, et al. An organization‐ and category‐level comparison of diagnostic requirements for mental disorders in Icd‐11 and Dsm‐5. World Psychiatry. 2021;20(1):34-51. 33432742 10.1002/wps.20825 PMC7801846

[17] Kilbourne AM, Beck K, Spaeth‐Rublee B, et al. Measuring and improving the quality of mental health care: a global perspective. World Psychiatry. 2018;17(1):30-8. 29352529 10.1002/wps.20482 PMC5775149

[18] Koike S, Uematsu A, Sasabayashi D, et al. Recent advances and future directions in brain MR imaging studies in schizophrenia: toward elucidating brain pathology and developing clinical tools. Magn Reson Med Sci. 2022;21(4):539-52. 34408115 10.2463/mrms.rev.2021-0050 PMC9618928

[19] Zhuo C, Li G, Lin X, et al. The rise and fall of Mri studies in major depressive disorder. Transl Psychiatry. 2019;9(1):335. 31819044 10.1038/s41398-019-0680-6 PMC6901449

[20] Iwashiro N, Suga M, Takano Y, et al. Localized gray matter volume reductions in the pars triangularis of the inferior frontal gyrus in individuals at clinical high-risk for psychosis and first episode for schizophrenia. Schizophr Res. 2012;137(1-3):124-31. 22425035 10.1016/j.schres.2012.02.024

[21] Horga G, Kaur T, Peterson BS. Annual Research Review: current limitations and future directions in MRI studies of child- and adult-onset developmental psychopathologies. J Child Psychol Psychiatry. 2014;55(6):659-80. 24438507 10.1111/jcpp.12185 PMC4029914

[22] Yamasaki S, Yamasue H, Abe O, et al. Reduced gray matter volume of pars opercularis is associated with impaired social communication in high-functioning autism spectrum disorders. Biol Psychiatry. 2010;68(12):1141-7. 20801427 10.1016/j.biopsych.2010.07.012

[23] Cross-Disorder Group of the Psychiatric Genomics Consortium. Electronic address: plee0@mgh.harvard.edu, Cross-Disorder Group of the Psychiatric Genomics Consortium. Genomic relationships, novel loci, and pleiotropic mechanisms across eight psychiatric disorders. Cell. 2019;179(7):1469-82.e11. 31835028 10.1016/j.cell.2019.11.020 PMC7077032

[24] Hettwer MD, Larivière S, Park BY, et al. Coordinated cortical thickness alterations across six neurodevelopmental and psychiatric disorders. Nat Commun. 2022;13(1):6851. 36369423 10.1038/s41467-022-34367-6 PMC9652311

[25] Ishida T, Nakamura Y, Tanaka SC, et al. Aberrant large-scale network interactions across psychiatric disorders revealed by large-sample multi-site resting-state functional magnetic resonance imaging datasets. Schizophr Bull. 2023;49(4):sbad022. 10.1093/schbul/sbad022 PMC10318885 36919870

[26] Xie C, Xiang S, Shen C, et al. A shared neural basis underlying psychiatric comorbidity. Nat Med. 2023;29(5):1232-42. 37095248 10.1038/s41591-023-02317-4 PMC10202801

[27] Nakamura Y, Ishida T, Tanaka SC, et al. Distinctive alterations in the mesocorticolimbic circuits in various psychiatric disorders. Psychiatry Clin Neurosci. 2023;77(6):345-54. 36905180 10.1111/pcn.13542 PMC11488596

[28] Nakamura Y, Okada N, Koshiyama D, et al. Differences in functional connectivity networks related to the midbrain dopaminergic system-related area in various psychiatric disorders. Schizophr Bull. 2020;46(5):1239-48. 31901932 10.1093/schbul/sbz121 PMC7505191

[29] Koike S, Sakakibara E, Satomura Y, et al. Shared functional impairment in the prefrontal cortex affects symptom severity across psychiatric disorders. Psychol Med. 2022;52(13):2661-70. 33336641 10.1017/S0033291720004742 PMC9647535

[30] Haas SS, Ge R, Agartz I, et al. 228 normative modeling of brain morphometry in clinical high-risk for psychosis. Biol Psychiatry. 2023;93(9):S185-6. 10.1001/jamapsychiatry.2023.3850 PMC10568447 37819650

[31] Bethlehem RA, Seidlitz J, White SR, et al. Brain charts for the human lifespan. Nature. 2022;604(7906):525-33. 35388223 10.1038/s41586-022-04554-y PMC9021021

[32] Howes OD, Kapur S. The dopamine hypothesis of schizophrenia: version III-the final common pathway. Schizophr Bull. 2009;35(3):549-62. 19325164 10.1093/schbul/sbp006 PMC2669582

[33] Wada M, Noda Y, Iwata Y, et al. Dopaminergic dysfunction and excitatory/inhibitory imbalance in treatment-resistant schizophrenia and novel neuromodulatory treatment. Mol Psychiatry. 2022;27(7):2950-67. 35444257 10.1038/s41380-022-01572-0

[34] Sander CY, Hesse S. News and views on in-vivo imaging of neurotransmission using pet and Mri. Q J Nucl Med Mol Imaging. 2017;61(4):414-28. 28750497 10.23736/S1824-4785.17.03019-9 PMC5916779

[35] Aryutova K, Stoyanov D. Pharmaco-magnetic resonance as a tool for monitoring the medication-related effects in the brain may provide potential biomarkers for psychotic disorders. Int J Mol Sci. 2021;22(17):9309. 34502214 10.3390/ijms22179309 PMC8430741

[36] Carter CS. Further evidence that Mri based measurement of midbrain neuromelanin may serve as a proxy measure of brain dopamine activity in psychiatric disorders. Neuropsychopharmacology. 2021;46(7):1231-2. 33526833 10.1038/s41386-020-00944-w PMC8134451

[37] Shibata E, Sasaki M, Tohyama K, et al. Use of neuromelanin-sensitive MRI to distinguish schizophrenic and depressive patients and healthy individuals based on signal alterations in the substantia nigra and locus ceruleus. Biol Psychiatry. 2008;64(5):401-6. 18452894 10.1016/j.biopsych.2008.03.021

[38] Shaw P, Gogtay N, Rapoport J. Childhood psychiatric disorders as anomalies in neurodevelopmental trajectories. Hum Brain Mapp. 2010;31(6):917-25. 20496382 10.1002/hbm.21028 PMC6870870

[39] Peyton L, Oliveros A, Choi DS, et al*.* Hippocampal regenerative medicine: neurogenic implications for addiction and mental disorders. Exp Mol Med. 2021;53(3):358-68. 33785869 10.1038/s12276-021-00587-x PMC8080570

[40] McHugo M, Armstrong K, Roeske MJ, et al*.* Hippocampal volume in early psychosis: a 2-year longitudinal study. Transl Psychiatry. 2020;10(1):306. 32873788 10.1038/s41398-020-00985-1 PMC7463254

[41] O’Neill A, Dooley N, Healy C, et al. Longitudinal gray matter development associated with psychotic experiences in young people. Biol Psychiatry Glob Open Sci. 2023;3(2):264-73. 37124352 10.1016/j.bpsgos.2022.02.003 PMC10140460

[42] Heilbronner U, Samara M, Leucht S, et al*.* The longitudinal course of schizophrenia across the lifespan: clinical, cognitive, and neurobiological aspects. Harv Rev Psychiatry. 2016;24(2):118-28. 26954596 10.1097/HRP.0000000000000092 PMC5079232

[43] Brain/MINDS. Objectives [Internet]. [cited 2023 Jun 27]. Available from: https://brainminds.jp/en/overview/objectives

[44] Koike S, Tanaka SC, Okada T, et al. Brain/MINDS beyond human brain MRI project: A protocol for multi-level harmonization across brain disorders throughout the lifespan. NeuroImage Clin. 2021;30:102600. 33741307 10.1016/j.nicl.2021.102600 PMC8209465

[45] Yamashita A, Yahata N, Itahashi T, et al. Harmonization of resting-state functional MRI data across multiple imaging sites via the separation of site differences into sampling bias and measurement bias. PLOS Biol. 2019;17(4):e3000042. 30998673 10.1371/journal.pbio.3000042 PMC6472734

[46] Koppe G, Meyer-Lindenberg A, Durstewitz D. Deep learning for small and big data in psychiatry. Neuropsychopharmacology. 2021;46(1):176-90. 32668442 10.1038/s41386-020-0767-z PMC7689428

[47] Oh J, Oh BL, Lee KU, et al. Identifying schizophrenia using structural MRI with a deep learning algorithm. Front Psychiatry. 2020;11:16. 32116837 10.3389/fpsyt.2020.00016 PMC7008229

